# Hydrogen Embrittlement of a T95 Low-Alloy Steel Charged by Electrochemical Method

**DOI:** 10.3390/ma18051047

**Published:** 2025-02-27

**Authors:** Luca Paterlini, Laura Vergani, Marco Ormellese, Arianna Curia, Giorgio Re, Fabio Bolzoni

**Affiliations:** 1 Dipartimento di Chimica, Materiali e Ingegneria Chimica “Giulio Natta”, Politecnico di Milano, 20131 Milan, Italy; luca.paterlini@polimi.it (L.P.); marco.ormellese@polimi.it (M.O.); arianna.curia@polimi.it (A.C.); grex94822@gmail.com (G.R.); 2 Dipartimento di Meccanica, Politecnico di Milano, 20156 Milan, Italy; laura.vergani@polimi.it

**Keywords:** hydrogen embrittlement, low-alloy steel, electrochemical charging, toughness, FEM, hydrogen diffusion coefficient

## Abstract

The hydrogen embrittlement of a typical Oil Country Tubular Good (OCTG) steel, API 5CT T95, was investigated through electrochemical hydrogen pre-charging followed by mechanical testing. J-integral and tensile tests were performed on electrochemically pre-charged samples, with varying charging conditions to simulate different hydrogen environmental exposure. Hydrogen concentration profiles during the electrochemical hydrogen charging process and subsequent mechanical testing in air were calculated with the support of hydrogen permeation tests and Finite Elements Method (FEM) mass diffusion analysis. This approach enabled a deeper understanding of the actual impact of hydrogen on the assessed mechanical properties. The results were compared with tests performed in air and with data available in the literature and were critically analyzed and discussed. A toughness reduction of up to 60% was observed under the most severe charging conditions; however, the alloy retained good ductility with a critical stress intensity factor of 124 MPa√m, well above the minimum values required for pipelines in high-pressure hydrogen gas and sour service applications, 55 MPa√m and 30 MPa√m, respectively, as specified by current ASME Standard and EFC Guidelines. Tensile tests on pre-charged specimens exhibited certain limitations due to the rapid hydrogen desorption rate with respect to the time required to conduct proper slow strain-rate tests.

## 1. Introduction

The energy share produced from renewable sources is steadily increasing and is bound to grow even further in the coming decades. Directive 2018/2001 [1], recently revised by the European Parliament, sets a target of up to 32 percent renewable energy production with respect to the total energy needs by 2030, and the target should be increased to full carbon neutrality for Europe in 2050 [2].

In the decarbonization process, hydrogen plays a key role as a low-carbon footprint energy carrier and as storage fuel for energy produced from renewable sources. The aim is to reduce the limitations related to the unreliability of these energy sources, which are highly dependent on unpredictable factors such as wind or solar coverage. To transport hydrogen over long distances, it could initially be blended with natural gas, Natural Gas (NG), up to 20% by volume within the existing transport and distribution network spread throughout the EU. A limit of 15–20% hydrogen in NG is, for example, indicated by CEN/TC 234 committee in CEN/TR 17797:2022 [3] to ensure those gas interchangeability criteria that are the basis of NG trade interchanges and that are necessary to minimize the impact on users of this “new” fuel.

An estimated 10^12^ m^3^ (108 tons) of hydrogen are already produced worldwide each year [4], mostly employed as chemical feedstock, produced by the chemical industry from methane or coal and used for ammonia synthesis, hydrogenation processes or other industrial purposes. Only a marginal fraction is produced from renewable sources and used as fuel.

In typical industrial applications, no issues are associated with pure hydrogen handling or any other hydrogen blend at room temperature, even when high pressure is involved. Hayden et al. [5,6], however, in their discussion on the new ASME B31.12 standard [7] published in 2009 and lastly revised in 2023, highlighted two very important facts: firstly, in the chemical industry, all the employed steels for hydrogen transport are low-grade steels, typically SA-106 Gr. B, API 5L X42 and X52, thus with a rather low yield strength (Specified Maximum Yield Strength-SMYS ≤ 52 ksi / 360 MPa); furthermore, the applied safety design factor is also low, on the order of 0.3–0.5 (30–50% SMYS).

When employed as an energy carrier, however, several issues different from those normally encountered in its industrial applications must be considered.

First, it must be noted that hydrogen in its gaseous phase carries about one-third of the NG energy per unit volume, keeping the other parameters involved constant. To compensate for this limitation, higher pressures or flow rates should be employed. To achieve this goal, it is then necessary to use steel alloys with higher SMYS, such as pipeline steels with grade higher than X70, in combination with high design factors.

A comprehensive energy supply system for a market with highly differentiated fuel requires the availability of articulated storage equipment, in terms of capacity and storage time, to compensate for the large differences between the fuel production rate and the market uptake rate. This is indeed essential for hydrogen, which is not readily available but has to be produced from other energy sources, specifically renewable ones that are inherently discontinuous and irregular. Storage needs to cover a wide range of performance, and for this purpose, both aboveground storage with limited capacity and shorter timespan (pressure tanks, cryogenic tanks for liquefied gas, etc.) and underground storage of larger capacity and longer term are available or under development [8].

The technologies proposed for underground storage are those already widely used for NG which are also currently under test as permanent storage for CO_2_ produced in Carbon Capture Utilization and Storage processes and consider the possibility of storage either in porous media (saline aquifers or depleted hydrocarbon reservoirs) or in engineered cavities [8,9,10].

On the other hand, regarding the materials required to build all the infrastructure necessary for the development of a hydrogen-based energy system, it is known that high-pressure hydrogen embrittles carbon and low-alloy steels [11,12], not developing specific failure phenomena, as for “sour” environments, but facilitating other fracture mechanisms such as fatigue or fracture under static load, when nucleated from a pre-existing defect.

To facilitate the employment of hydrogen as an energy carrier, it is therefore necessary to deepen the understanding of the behavior of metallic materials in the presence of high-pressure hydrogen throughout the whole supply chain, from production to its final use. Long-distance transport, storage, and accumulation stages are particularly relevant for their high operative pressure.

Regarding pipeline steels, in the preparation phase of the ASME B31-12 standard, an extensive characterization campaign was conducted from the point of view of their toughness and fatigue behavior in a hydrogen environment using fracture mechanics methods by measuring the hydrogen fracture toughness (*K_JcH_*) parameter and the fatigue crack growth rate, i.e., the curve da/dN-ΔK [13,14]. Nanninga et al. conducted a review focusing on the fatigue crack growth response of pipeline steels exposed to gaseous hydrogen environments [15]. Additionally, Slifka et al. investigated the effects of hydrogen pressure and loading frequency on crack growth rates [16], while Amaro et al., in their early work, proposed a predictive fatigue crack growth model calibrated on API X100 and X52 steels. Their study emphasized the need for engineered models to ensure the safe transportation and distribution of hydrogen [17]. Furthermore, Briottet et al. provided a comprehensive comparison of various mechanical testing methods used to quantify hydrogen effects, including tensile tests, disk pressure tests, fracture toughness and fatigue crack growth measurements, as well as WOL (Wedge Opening Load) tests. These tests were conducted in both neutral atmospheres and high-pressure hydrogen gas environments [18].

Other relevant results have been obtained more recently by DVGW [19] through the characterization in terms of K_IH_ and fatigue crack growth rate of as many as 26 different pipeline steels currently used in Germany and Europe for NG transportation, covering the production years from 1930 to 2022, nominal yield strength ranging from 207 to 550 MPa, and widely varying microstructures covering the evolutionary history of pipeline steels over the past 90 years. For each steel, base metal was tested. Additionally, longitudinal or spiral pipe production weld or circumferential field joint weld were occasionally tested, mainly weld metal and in a few cases, heat affected zone. In the same project, six valve materials were also evaluated.

In the field of hydrogen storage, extensive information is already available on materials suitable for traditional technologies, such as high-pressure gas storage in vessels or liquefied hydrogen storage in cryogenic tanks. However, data on the behaviour of OCTG steels, specifically those classified under the API 5CT specification, in high-pressure hydrogen environments remain limited. These steels have potential applications for casing and tubing in large-volume underground hydrogen storage projects.

Currently, only a few studies have examined the compatibility of these steel alloys with hydrogen environments. Among the most relevant is the work of Cheng et al., which investigated the tensile behaviour of API 5CT L80 and API 5CT P110 in the presence of hydrogen, demonstrating their susceptibility to hydrogen embrittlement, as evidenced by a significant reduction in elongation at break [20].

Therefore, this study focuses on the characterization of API 5CT T95 Type 1 steel, which is widely used for casing and tubing in oil and gas production. This steel was selected due to its proven performance in sour environments, which is already well documented in the literature.

The T95 has one of the highest SMYS values among those in the API 5CT specification (SMYS = 95 ksi = 655 MPa; UTS_min_ = 110 ksi = 724 MPa) and as far as composition is concerned, it is a low-alloy Cr-Mo steel whose composition (C = 0.35% max, Cr = 0.4–1.5%, Mo = 0.25–0.85%) is not comparable with that of ASTM Cr-Mo steels used for pressure vessels and cylinders e.g., compared to 34CrMo4 steel (approximately equivalent to AISI 4135), widely used in Europe for high-pressure hydrogen cylinders, the composition range of Cr and Mo is much wider and the Mo/Cr ratio is higher.

This paper proposes a method for evaluating the susceptibility of steels to hydrogen embrittlement by employing pre-charging followed by mechanical testing. While this approach offers a cost-effective alternative to in-situ testing, conducting mechanical tests on pre-charged specimens presents certain limitations in assessing the validity of the results. Specifically, hydrogen concentration and distribution within the specimens during both the charging and mechanical testing phases remain unknown variables, often overlooked.

To address these limitations, a series of preliminary tests was conducted to analyse the material’s response to electrochemical charging and to determine key parameters such as the hydrogen diffusion coefficient and solubility. Based on the acquired data, a Finite Element Method (FEM) analysis was performed to calculate and predict the hydrogen concentration across the sample thickness during both the charging and mechanical testing phases. This approach provided a solid foundation for selecting optimal pre-charging conditions and an appropriate testing rate to minimize excessive hydrogen desorption.

## 2. Materials and Methods

### 2.1. Material and Preliminary Characterization

The material analyzed in this paper is an OCTG API 5CT T95 Type 1 steel, taken from a pipe with diameter D = 473 mm and thickness t = 14.7 mm, supplied by ENI. The chemical composition, measured through Glow Discharge Optical Emission Spectroscopy, is given in Table 1. The component was subjected to quench and temper heat treatment in accordance with the API 5CT standard requirements [21] (minimum tempering temperature T = 649 °C).

**Figure 1:**
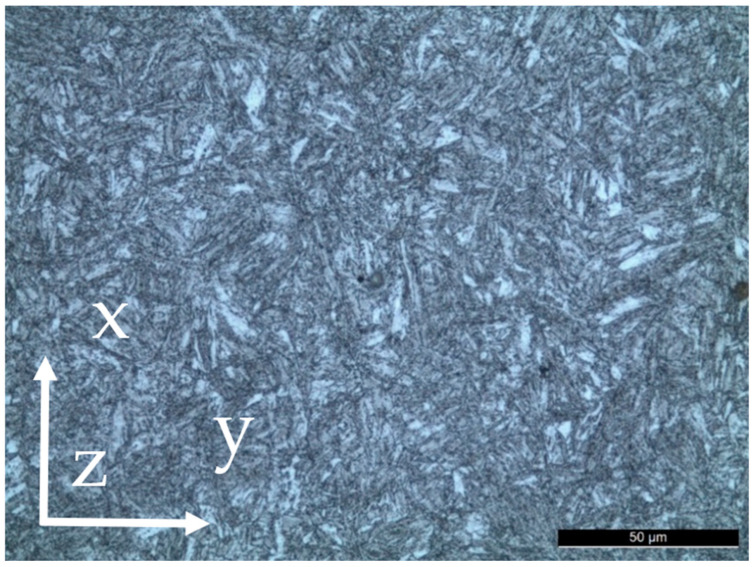
API 5CT T95 steel. Microstructure, 5% Nital etching, Optical Microscope x500.

**Figure 2:**
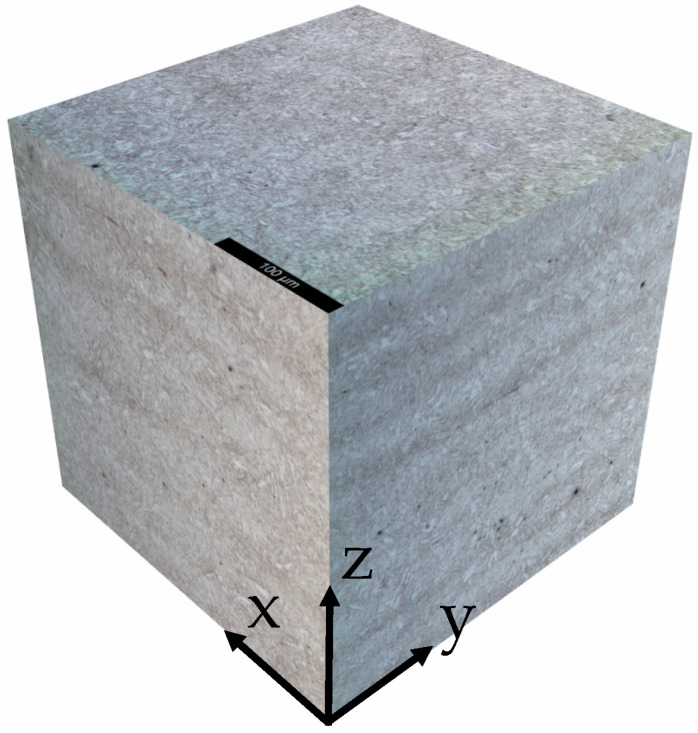
API 5CT T95 steel. Microstructure on the three principal planes, Optical Microscope x200.

The material was characterized in the as-received condition. From the micrographic analysis (Figure 1), it appears that the microstructure is acicular fine-grained tempered martensite; banding is also visible (Figure 2), as a consequence of the plastic deformation process to which the material was subjected during its manufacturing. The results of Vickers hardness tests performed along the three main directions of the pipe are shown in Table 2. The observed microstructure and hardness values are consistent with the chemical composition and heat treatment required by the standard [21].

**Figure 3:**
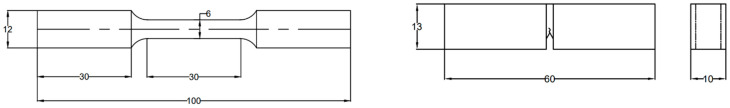
Drawings of the cylindrical specimens for monotonic tensile tests (**left**) and SEB pre-cracked specimens for J-integral tests (**right**). Dimensions in mm.

The mechanical properties at room temperature were measured in air through monotonic tensile tests on cylindrical specimens and J-integral tests on Single Edge Bend (SEB) specimens. Sample dimensions are given in Figure 3. The SEB specimen notches were obtained by means of electro-erosion technique and the pre-crack by applying sinusoidal variable loading. After the pre-crack reached the length required by the standard, lateral side grooves were cut on the specimens. Side grooves help the crack propagation inducing a triaxial stress state. All samples were sandpaper polished up to P600 grit.

The tensile tests were performed according to ASTM A370 [22] by using an MTS 100 kN testing machine (MTS Systems Corporation, Eden Prairie, MN, USA) with an extensometer with 25 mm measuring base. The tests were carried out in displacement control and the measured parameters are Yield Strength (YS), Ultimate Tensile Strength (UTS), Elongation at break (El) and Reduction Area (RA).

The toughness tests were performed according to ASTM E1820 [23] Standard using a servo-hydraulic MTS machine (MTS Systems Corporation, Eden Prairie, MN, USA) and a displacement gage. 

Three specimens were tested for both tensile and fracture toughness tests; the results are provided in Table 3 and Table 4, respectively. The *K_Jc_* value reported in Table 4 is derived from the strain energy release rate (*J_Q_*) parameter through the following equation:

(1) $$K_{Jc}=\sqrt{\frac{E\cdot J_{Q}}{1-\nu^{2}}}$$

where the value of 212 GPa was adopted for the Young’s modulus, *E*, of the steel, which is the average value measured in the tensile tests, and a value of 0.27 for the Poisson modulus ν.

### 2.2. Hydrogen Diffusion Coefficient Measurement

Hydrogen diffusion coefficient *D_H_* of API 5CT T95 at 20 °C was measured with a Devanathan-Stachurski electrochemical diffusion cell, operated according to ISO-17081, using the Time-lag equation for its calculation [24]:

(2) $$D_{H}=\frac{L^{2}}{6t_{lag}}$$

(3) $$t_{lag}=time\;to\;achieve\;a\;value\;of\;\frac{J_{\left(t\right)}}{J_{SS}}=0.63$$

where:

- *L* is the sample thickness in (m);
- *J*_(*t*)_ is the time-dependent atomic hydrogen permeation flux as measured on the oxidation side of the sample;
- *J_SS_* is the atomic hydrogen permeation flux at steady-state as measured on the oxidation side of the sample.

For the transient measurements, a 0.1 mol NaOH solution (pH 13) was employed as electrolyte in both the cathodic and the anodic side. The anodic side of the sample was pre-passivated at +340 mV Ag/AgCl reference electrode in the NaOH solution for 24 h, to achieve full passivation and low background current, below 50 nA/cm^2^. Upon reaching complete passivation on the anodic side, the cathodic chamber was rapidly flooded with nitrogen-purged soda solution, and a cathodic current of 2 mA/cm^2^ was imposed with a galvanostat on the cathodic side of the sample, for the whole duration of the tests. The oxidation current during the permeation transient was recorded with a datalogger. To obtain data consistency, four permeation tests were performed on four different specimens.

### 2.3. Electrochemical Hydrogen Pre-Charging

To simulate the hydrogen charging due to high-pressure hydrogen gas, test specimens were electrochemically pre-charged in 1N H_2_SO_4_ + 250 mg/L As_2_O_3_ solution, commonly employed in the literature [25,26,27,28,29,30,31]. The complete electrochemical charging procedure is thoroughly described by Bolzoni et al. in [32,33].

Preliminary electrochemical charging tests were carried out on unnotched SEB specimens, with the same dimensions as the SEB samples shown in Figure 3. The purpose of these tests was to investigate the influence of current density and charging time on the hydrogen concentration within the specimen and to assess the susceptibility of the steel to hydrogen-induced damage during electrochemical charging.

The preliminary electrochemical charges were performed in galvanostatic mode, applying current densities of 0.5 mA/cm^2^ and 5 mA/cm^2^ with an AMEL 2049 potentiostat (AMEL srl, Milan, Italy), for a duration ranging between 4 and 72 h.

The absorbed diffusible hydrogen, not irreversibly trapped inside the specimen, was measured through hot glycerol extraction, at a temperature of 150 °C. The measurements were made right at the end of the electrochemical charging, after water rinsing and a subsequent drying with acetone. The hot glycerol extraction measurement is widely employed in the literature despite its rather low precision [34,35,36], because with this technique it is possible to measure hydrogen content in bulky specimens (>50 g) [33], as the ones employed in this study.

### 2.4. FEM Analysis of Hydrogen Concentration

The *D_H_* value of the API 5CT T95 steel measured with the permeation method was employed to estimate through FEM analysis the hydrogen concentration distribution inside the samples, during the electrochemical charge and during the execution of the mechanical tests.

The FEM model employed to calculate the hydrogen concentration was created with the support of COMSOL Multiphysics^©^ 5.5 software. The model obeys Fick’s diffusion laws, according to the convection-diffusion equation in absence of advective flux and net volumetric sources:

(4) $$\frac{\partial \varphi }{\partial t}=D\nabla^{2}\varphi$$

where:

- φ is the local hydrogen concentration, in (ppm);
- t is the time, in (s);
- *D* is the diffusion coefficient in (m^2^/s).

The only driving force in the FEM simulation is the hydrogen concentration gradient within the sample, which was modelled with identical dimensions to the corresponding real samples (Figure 3). To simplify the computation step, two reasonable approximations were adopted:

- The diffusion coefficient within the material was set as constant throughout its volume in any direction (homogeneous and isotropic material); The material can reasonably be modelled as isotropic in first approximation, as only slight banding was detected during the micrographic analysis.
- The hydrogen concentration over the entire surface of the sample is constant over time and equal in every point (homogeneous hydrogen surface concentration conditions). This approximation can be safely assumed, as subsurface hydrogen concentration is determined by cathodic current density during electrochemical charge, and the high conductivity of the 0.5 mol/l H_2_SO_4_ solution, equal to 20 S/m [37], ensures a smooth current density over the whole specimen surface reducing the ohmic drop in the electrolyte.

A constant hydrogen concentration (*C*_0_) was set on the whole sample surface as boundary condition, while the initial hydrogen bulk concentration (*C_B_*) on the whole sample volume was set equal to 0 at the beginning of the simulation.

The *D_H_* of the material was set equal to the average *D_H_* measured according to ISO 17081, as previously described (Section 2.2). The mesh was built with tetrahedral elements with maximum dimension of 0.2 mm, for a total of about 1.2 × 10^6^ elements.

### 2.5. Mechanical Tests on Charged Samples and Fractography

Since it was impossible to measure the absorbed hydrogen and perform a mechanical test on the same sample, an API 5CT T95 block of the same geometry as the mechanical test specimens was always added during each electrochemical charge of the mechanical test specimens, intended for the measurement of the absorbed hydrogen. The hydrogen concentration measured in the test samples was then compared with the hydrogen concentration measured on each specimen at the end of the mechanical test to confirm the pre-charge effectiveness and to estimate the hydrogen desorption during the test itself.

For the mechanical tests, two cylindrical specimens were electrochemically pre-charged at 0.5 mA/cm^2^ for 24 h and a total of four SEB specimens were pre-charged, two at 0.5 mA/cm^2^ and two at 5 mA/cm^2^ for 48 h, with the goal of simulating two different conditions of hydrogen exposure conditions, as described in Section 2.3. Immediately after the charging, the samples were stored in liquid nitrogen at −196 °C to reduce hydrogen desorption. The mechanical tests were performed at room temperature (18–22 °C). The geometry of tensile specimens is reported in Figure 3. The elongation rate of the test was reduced to 0.4 mm/min for hydrogen-charged specimens. This elongation rate corresponds to a strain rate of 2.2 × 10^−4^ s^−1^, which is higher than that usually adopted for slow strain tests of 10^−5^ s^−1^. Slower strain rates are usually associated with higher embrittlement when equal environmental conditions are applied, as in the case of in situ tests. However, the selected strain rate was a calculated compromise between the need to give more time to the hydrogen to diffuse towards the plastic deformation area of the specimen during the test and that of limiting its desorption from the specimen. The test duration was estimated through FEM analysis so that the average bulk hydrogen concentration in the specimen at the end of the test was at least 50% of the initial concentration. Cheng et al., when testing two Q&T low-alloy steels through electrochemical pre-charging techniques, faced similar desorption issues, applying a strain rate of 1 × 10^−3^ and obtaining elongation at break reduction consistently with the applied hydrogen charging conditions [20].

The toughness tests were performed with the same specimen geometry and the testing procedure used for the tests without hydrogen. The test speed was imposed so that the test lasted about 1 h, a time considered as the optimal compromise to the needs already highlighted for the tensile test.

Immediately after the test, the specimens for the toughness measurement were again immersed in liquid nitrogen, and then the residual hydrogen was measured using the glycerol method mentioned earlier. Lastly, the samples were heat tinted, fractured in liquid nitrogen and a fractographic analysis with SEM was performed to evaluate the effects of hydrogen on the fracture morphology.

## 3. Results

### 3.1. Hydrogen Diffusion Coefficient

**Figure 4:**
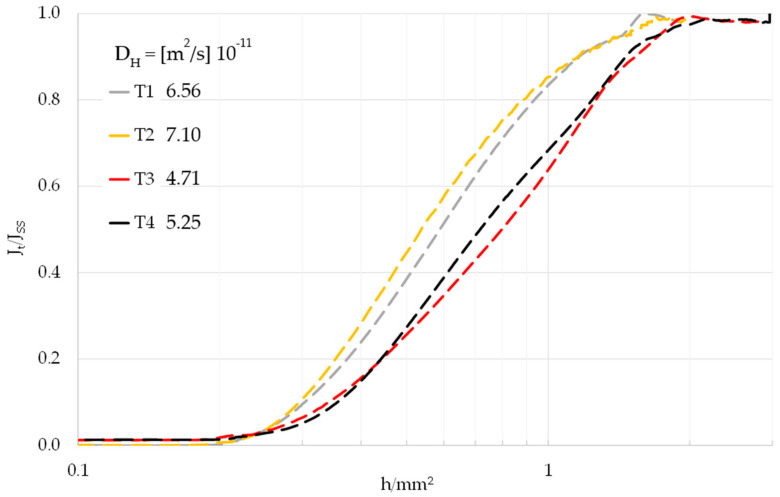
API 5CT T95 hydrogen permeation transients measured with Devanathan-Stachurski permeation cell. Time normalized with respect to thickness on X axis, normalized current density on Y axis.

To achieve data consistency, four hydrogen permeation transients were performed on samples between 1.8 mm and 2.0 mm thick. The four transients are shown in Figure 4. The hydrogen diffusion coefficient, determined using the Time-lag method (Equation (2)), was consistent across the tests, with an average value of *D_H_* = 5.9 m^2^/s 10^−11^, which is in excellent agreement with diffusion coefficients in the literature for quenched and tempered steels with comparable microstructure and chemical composition [38,39,40,41].

### 3.2. Electrochemical Hydrogen Preliminary Charges

**Figure 5:**
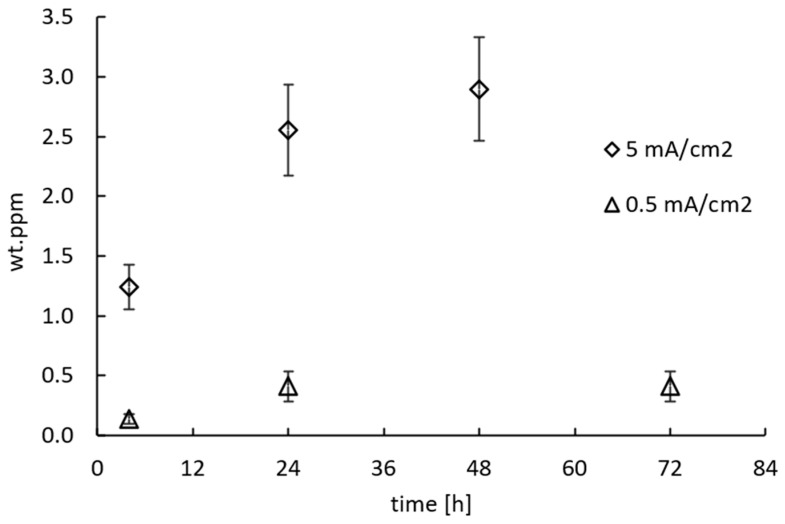
Preliminary hydrogen uptake tests, 0.5 mA/cm^2^ and 5 mA/cm^2^. Samples dimension: 60 × 13 × 10 mm.

The results of the preliminary charging tests are reported in Figure 5. No blisters were observed on the samples surface after the electrochemical charging, even when the harsher charging conditions were applied (5 mA/cm^2^ for 48 h). Increasing current density from 0.5 mA/cm^2^ to 5 mA/cm^2^ resulted in an increase of absorbed hydrogen from 0.3 ppm to 2.6 ppm after a 24 h electrochemical charge, almost an order of magnitude. Trautmann et al. [42,43,44] state that for martensitic microstructures hydrogen concentrations ranging between 0.1 and 0.5 ppm are commonly achieved in high pressure hydrogen environment, up to 10 MPa, while higher concentrations, in the order of few ppm, are typically found when working in sour environments [44]. Accordingly, the electrochemical hydrogen charge with 0.5 mA/cm^2^ was considered representative of high-pressure hydrogen for pipeline applications while 5 mA/cm^2^ more representative of mildly sour applications.

### 3.3. Hydrogen Concentration Profiles

**Figure 6:**
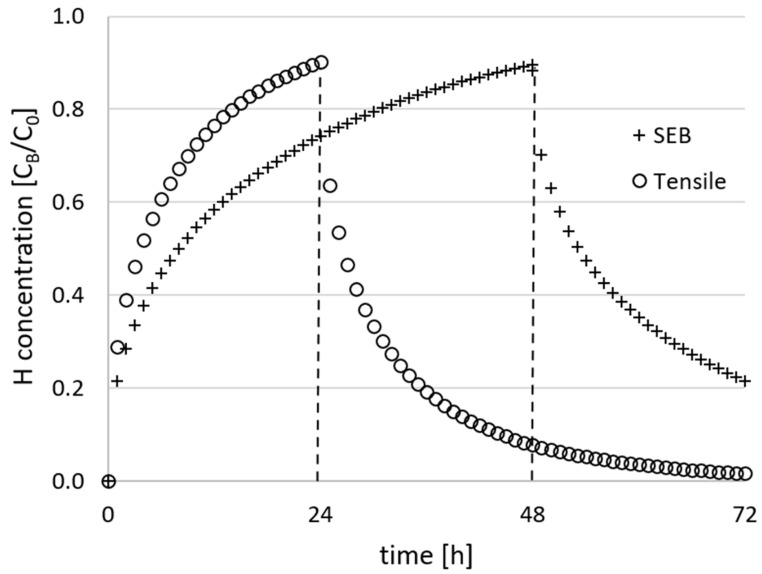
Normalized hydrogen concentration during the electrochemical hydrogen charge and desorption phase, calculated with FEM analysis. The vertical black lines identify the tensile and SEB samples actual pre-charge duration and the onset of the desorption simulation. *D_H_* = 5.9 × 10^−11^ m^2^/s.

**Figure 7:**
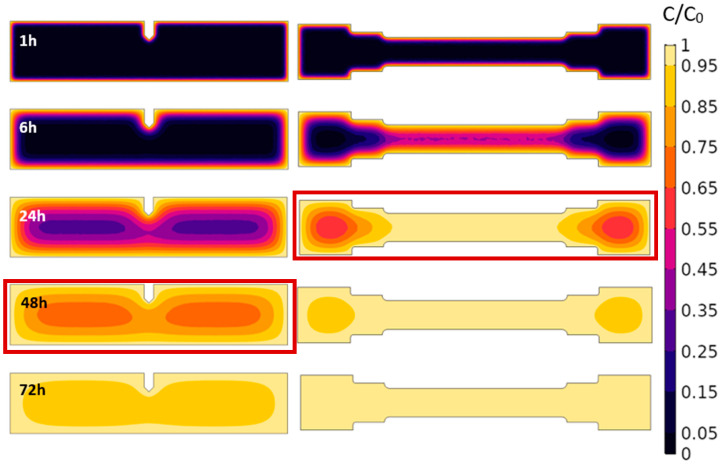
Normalized hydrogen concentration in SEB and tensile samples exposed to electrochemical charge for 1 h, 6 h, 24 h, 48 h and 72 h. *D_H_* = 5.9 × 10^−11^ m^2^/s. The applied pre-charge durations are highlighted in red boxes.

According to the FEM diffusion simulation described in Section 2.4, the average normalized hydrogen concentration as function of time during the electrochemical charge of the tensile and SEB samples (dimensions in Figure 3) are reported in Figure 6. The calculated hydrogen concentration distribution inside the samples as function of time during the electrochemical charge is depicted in Figure 7. The hydrogen concentration inside the sample was considered homogeneous enough to perform mechanical tests when:

(5) $$C_{B}=0.9*C_{0}$$

where:

- *C_B_* is the average bulk hydrogen concentration during the electrochemical charge;
- *C*_0_ is the hydrogen surface concentration.

This condition, according to the diffusion FEM analysis depicted in Figure 6, was satisfied for *t* ≥ 22 h for tensile samples and for *t* ≥ 47 h for SEB samples. Consequently, tensile samples were electrochemically pre-charged for 24 h prior to the mechanical tests, while the SEB samples, slightly thicker, for 48 h. The hydrogen concentration profiles of SEB and tensile specimens obtained at the end of the hydrogen charge, respectively after 48 h and 24 h, are visualized in Figure 7.

**Figure 8:**
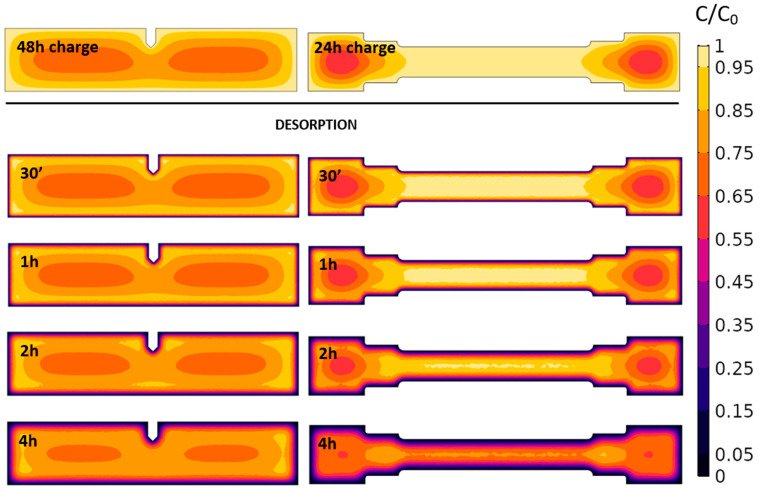
Normalized hydrogen concentration in SEB and tensile samples exposed, respectively, to 48 h and 24 h of electrochemical charge. Hydrogen concentration after 30′, 1 h, 2 h and 4 h desorption. *D_H_* = 5.9 × 10^−11^ m^2^/s.

After 48 h of charging, the central regions of the SEB specimens do reach a *C* > 0.65 *C*_0_ according to the FEM analysis performed, ensuring a rather smooth hydrogen concentration profile along the crack propagation path. Similarly, after 24 h of pre-charge, the hydrogen concentration in the middle section of the tensile specimen is completely homogeneous (*C* > 0.95 *C*_0_ across the whole thickness), as displayed in Figure 7. Furthermore, the hydrogen distribution inside the specimens calculated during the first four hours of desorption is displayed in Figure 8, highlighting the consistency over time of the hydrogen concentration in the first few hours, during which the mechanical tests are performed. During the execution of the toughness test, the crack-tip propagates toward the core of the pre-charged specimen, moving toward the effective hydrogen reservoir, buffering the effect of the hydrogen desorption from the sample surface.

### 3.4. Tensile Tests

The tensile tests performed on pre-charged cylindrical samples returned the results reported in Table 5. No evident changes were observed on the YS and the UTS parameters, as abundantly reported in the current literature regarding carbon and low alloy steels [45,46,47,48,49,50,51]. However, a remarkable reduction of the ductility-related parameter El (elongation at break) was registered, as well as a slight decrease of reduction of area (RA).

For all the measured parameters except RA, the effect of hydrogen was evaluated through the “embrittlement index” defined as *I_X_ = (X*_0_ *− X_H_)/X*_0_, where *X* is the selected parameter, *X*_0_ is its value measured on the material as received, and *X_H_* is its value measured on the material pre-charged with hydrogen.

For the RA parameter, on the other hand, in accordance with the standard ANSI/CSA CHMC 1 [52], the ratio of the reduction of area, RRA, was adopted:

*RRA* = *RA_H_*/*RA_air_* (6)

where:

- *RA_H_* is the reduction of area in the presence of hydrogen;
- *RA_air_* is the reduction of area in the absence of hydrogen.

The elongation at break (El), measured according to ASTM A370 [22], which is closely related to the ductility of the material, was reduced by 31% (*I_El_* = 0.31) in the hydrogen pre-charged specimens compared to those tested as received, while RA, which is usually the most sensitive parameter to the presence of hydrogen during tensile tests [13,44,45,46], undergoes a modest reduction to the limits of statistical significance resulting in *RRA* = 0.96. It is therefore concluded that, with the material and under the experimental conditions adopted in the present research, plastic instability arises first in the presence of hydrogen but necking is not significantly modified.

The small impact of hydrogen on the *RA* parameter may be attributed both to the limited hydrogen content in the specimens and to the previously mentioned fact that the applied strain rate may not be sufficiently low. However, under the present experimental conditions, the reduction in elongation (El) demonstrates that T95 steel is sensitive to the presence of hydrogen, even at low concentrations, such as the 0.3 ppm introduced by the charging conditions applied to the tensile test specimens.

### 3.5. Toughness Tests

**Figure 9:**
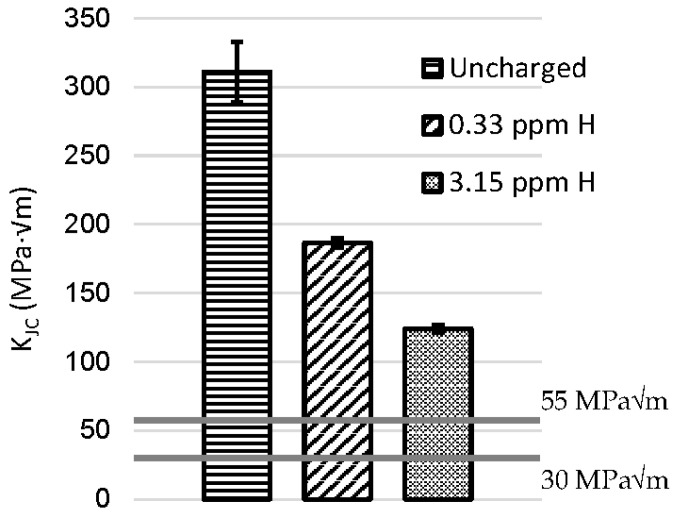
*K_Jc_* measured by three-point bending tests on uncharged samples and pre-charged samples with two different hydrogen contents. Horizontal grey line indicates the minimum *K_Jc_* value required by ASME B31-12 [7] for pipeline applications.

**Figure 10:**
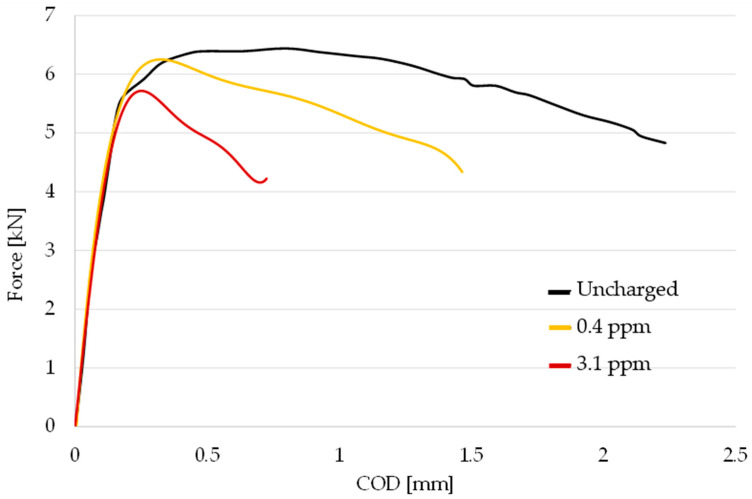
Load-crack mouth displacement plots acquired during the J-integral testing of hydrogen charged samples; Uncharged condition, 0.4 ppm hydrogen concentration and 3.1 ppm hydrogen concentration.

The results of the J-integral tests performed on the charged SEB specimens and the respective charging conditions are summarized in Table 6 and visualized in Figure 9. Load-crack mouth displacement plots acquired during the toughness testing of both hydrogen charged and uncharged specimens are reported in Figure 10. The fracture exhibits an earlier transition to plastic behavior, consistent with the increase in hydrogen content. Nevertheless, the material retains a moderate level of plasticity, preventing the occurrence of catastrophic brittle fracture during the toughness test.

The effect of hydrogen on toughness is clearly visible in both charging conditions, with a neat reduction of the *K_Jc_* parameter. The embrittlement index for the stress intensity factor reduction is:

(7) $$I_{K}=\frac{K_{Jc}-K_{JcH}}{K_{Jc}}$$

as previously defined.

When the lower cathodic current density was employed during the pre-charging with an estimated hydrogen content equal to 0.4 ppm, *K_JcH_* decreased to an average 186 MPa√m, with rather small scatter between the two samples tested in the same condition. The average embrittlement index in these environmental conditions was *I_K_* = 0.40.

The toughness decreases even further with the increasing severity of the electrochemical pre-charge applied and a higher hydrogen content equal to 3.1 ppm, dropping to an average *K_JcH_* = 124 MPa√m, displaying an embrittlement index I_K_ = 0.60. Although the reduction is severe, even in the harsher conditions applied, the measured *K_JcH_* is well above the minimum required value specified by the current standards for pipeline [7] and guidelines for sour application (Guidelines on Materials Requirements for Carbon and Low Alloy Steels For H_2_S-Containing Environments in Oil and Gas Production) [53], equal to 55 MPa√m and 30 MPa√m, respectively (highlighted by the grey horizontal lines in Figure 9), and is enough to withstand eventual defects which may form during its employment.

The hydrogen charged J-integral samples spend about 90 min at room temperature, a consequence of the time required for their conditioning to room temperature (~10–15 min), to perform the mechanical tests (~45–60 min) and the time required to dismount the sample (~15 min). The samples were stored in liquid nitrogen for all the residual time. Therefore, the residual hydrogen content measured at the end of the whole testing procedure accounts for all these contributions. The hydrogen concentration measured within the SEB samples after the mechanical test was always equal to or greater than 42% of the tester concentration measured before the toughness test (Table 6).

Consequently, it can be assumed that the sample bulk acts as an effective hydrogen reservoir throughout the mechanical test run, ensuring adequate hydrogen flow to the crack-tip.

### 3.6. Fractographic Analysis

#### 3.6.1. Tensile Samples

**Figure 11:**
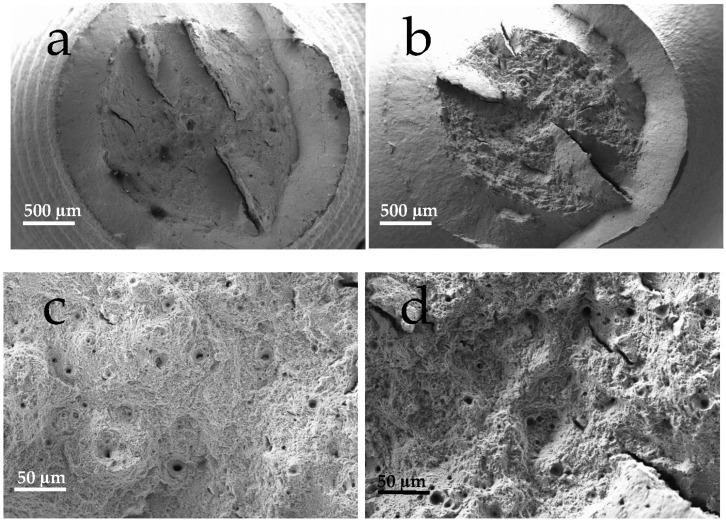
Fractographic analysis of cylindrical tensile samples: (**a**) uncharged sample, 100X; (**b**) charged sample, 100X; (**c**) dimples on uncharged sample, 1000X, (**d**) dimples on charged sample, 1000X.

Fractographic analysis of the hydrogen-free steel tensile specimens (Figure 11a,b) shows that macroscopically fracture exhibits the typical cup-cone appearance with fracture components perpendicular to the main fracture, along the x-y plane of Figure 2 and parallel to the banding evidenced by metallographic analysis. The fracture nucleation in the specimens always occurred in the central zone where the stress triaxiality intensifies. Microscopically, the fracture surface is very smooth and displays very fine dimples with some larger ones nucleated around non-metallic inclusions. In the hydrogen-charged specimens, a cup-cone fracture also occurs with perpendicular components in the same plane as already described for the hydrogen-free specimens (Figure 11c). The fracture nucleates in the central part of the specimen in the zone that retains the higher hydrogen concentration over time and where the triaxiality of the stresses promotes the diffusion of the pre-charged hydrogen. Moreover, there was no evidence of crack nucleation on the outer surface where the diffusion towards the outer surface rapidly reduces the hydrogen availability during the test. The fracture in the central zone of the specimen with hydrogen appears coarser than without hydrogen and on a microscopic level (Figure 11d).

#### 3.6.2. SEB Samples

**Figure 12:**
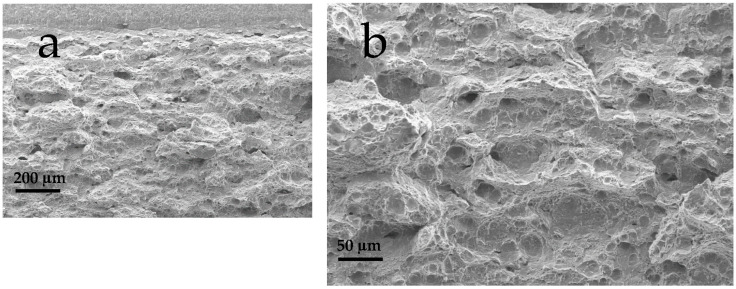

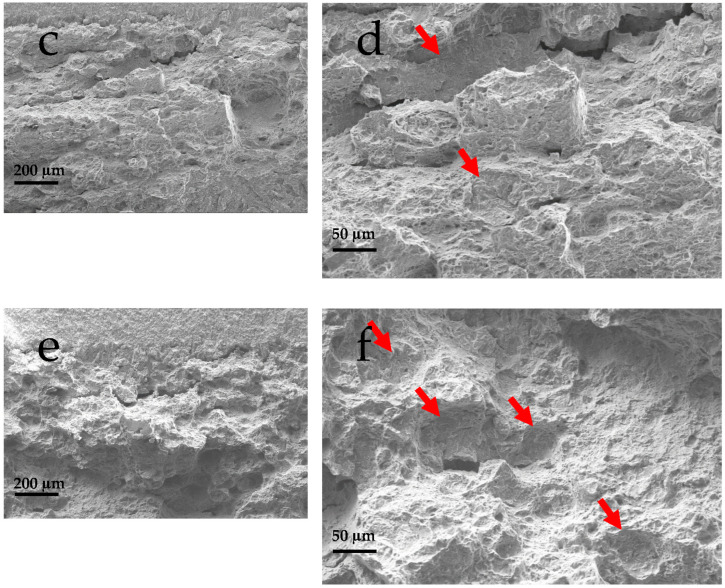
Fractographic analysis of uncharged SEB: (**a**) fatigue-tearing region interface, 200X; (**b**) tearing region, 500X; SEB, 0.4 ppm hydrogen: (**c**) fatigue-tearing region interface, 200X; (**d**) mixed ductile and quasi-cleavage region, 500X; SEB, 3.1 ppm hydrogen: (**e**) fatigue-tearing region interface, 200X; (**f**) prevalent quasi cleavage region, 500X. Red arrows highlight brittle quasi cleavage regions.

The uncharged SEB sample displays a fully ductile micro-voids fracture morphology (Figure 12a,b), which can be visualized by the typical formation of ductile dimples nucleated around non-metallic inclusions. With the introduction in the lattice of few tenths of ppm of hydrogen, the *K_JcH_* decreased to 60% of its original value, confirmed by the appearance of isolated regions where both the micro-voids formation and the quasi-cleavage mechanisms occurred in competition (Figure 12c,d). Moreover, small transverse brittle cracks appear on the main fracture surface. Increasing furthermore the hydrogen concentration pre-charged in the SEB sample, the fracture morphology in the tearing region still appears as competition between a ductile micro-void and a brittle quasi-cleavage fracture morphology (Figure 12e,f), although the brittle portion increases, accordingly with the further toughness reduction. Apparently brittle transverse microcracks are also present in samples with higher hydrogen content.

## 4. Conclusions

Electrochemical hydrogen pre-charging was employed to study the behaviour of an API 5CT T95 low-alloy steel in a rich hydrogen environment, in two different exposure conditions.

- Electrochemical hydrogen charging tests proved that in 1N H_2_SO_4_ + 250 mg/L As_2_O_3_ solution high-pressure hydrogen and mildly sour environments can be simulated applying a cathodic current density of, respectively, 0.5 mA/cm^2^ and 5 mA/cm^2^;
- During the toughness mechanical tests performed at room temperature, the hydrogen concentration never dropped below 42% of its initial value;
- The SEB specimens pre-charged with 0.4 and 3.1 ppm of hydrogen displayed a significant drop in toughness, with an embrittlement index *I_K_* = 0.40 and *I_K_* = 0.60, respectively. Nevertheless, the material retained a partially ductile behavior even when the most severe environmental conditions were applied, and the lower registered *K_JcH_* = 124 MPa√m is well above the minimum value of 55 MPa√m and 30 MPa√m, required by ASME B31.12 [7] Standard and EFC Guidelines [53];
- The toughness reduction in hydrogen pre-charged specimens was confirmed by the appearance of brittle regions characterized by quasi-cleavage morphology;
- Tensile tests showed a modest effect of hydrogen only on ductility parameters (RA, El), so hydrogen pre-charging may not be the correct testing methodology for thin specimens, as consistent hydrogen desorption takes place when slow strain rates are applied.

Further testing on different steel alloys belonging to the O&G field will be needed to understand the representativeness of hydrogen pre-charging as methodology to study high-pressure hydrogen induced embrittlement and eventually extend it to sour environments.

## Figures and Tables

**Table 1:** API 5CT T95 steel. Chemical composition.

Steel	C (%)	Mn (%)	Si (%)	S (%)	P (%)	Cr (%)	Mo (%)	Ni (%)
T95	0.342	0.358	0.234	<0.001	0.011	1.191	0.798	0.161

**Table 2:** API 5CT T95 steel. Average Vickers hardness on the three principal planes.

Plane	X-Y	X-Z	Z-Y
HV_30_	263 ± 9.7	257 ± 6.1	259 ± 3.5

**Table 3:** API 5CT T95 steel. Average YS, UTS, El and RA values measured by monotonic tensile tests performed in air at room temperature.

	YS (MPa)	UTS (MPa)	El (%)	RA (%)
Average	702 ± 1.5	806 ± 2.3	24.9 ± 0.1	76.8 ± 0.8

**Table 4:** API 5CT T95 steel. Average *J*_q_ and *K_J_*_c_ values measured by J-integral tests performed on SEB specimen in air at room temperature.

	*J_Q_* (N/mm)	*K_Jc_*$\;(\mathrm{MPa}\sqrt{m}$)
Average	448 ± 66	311 ± 22

**Table 5:** Pre-charging conditions, hydrogen content measured before the tensile test. Tensile tests results.

	Cath. i (mA/cm^2^)	H Conc. (ppm)	YS (MPa)	UTS (MPa)	El (%)	I_El_	RA [%]	RRA
Average	0.5	0.3	705 ± 5.7	797.5 ± 7.8	17.3 ± 1.6	0.31 ± 0.06	73.8 ± 0.6	0.96 ± 0.01

**Table 6:** Pre-charging conditions, hydrogen content measured before and after the toughness test. Toughness tests results.

	Cath. i (mA/cm^2^)	Initial H Conc. (ppm)	H Conc. After Test (ppm)	*J_Q_* (N/mm)	*K_JcH_* (MPa√m)	*I_K_*
Average	0.5	0.4	0.17 ± 0.0	159 ± 5.7	186.5 ± 3.5	0.40 ± 0.1
	5	3.1	1.98 ± 0.1	72.1 ± 0.0	124.2 ± 2.8	0.60 ± 0.07

## Data Availability

The original contributions presented in the study are included in the article, further inquiries can be directed to the corresponding author.

## Nomenclature

ν	Poisson coefficient
φ	Local hydrogen concentration
C_B_	Bulk hydrogen concentration
C_0_	Hydrogen subsurface concentration
D	Diffusion coefficient
D_H_	Apparent hydrogen diffusion coefficient
El	Elongation at break
I_El_	Embrittlement index for the elongation at break
I_K_	Embrittlement index for the toughness reduction
J_Q_	Strain energy release rate
J_SS_	Atomic hydrogen permeation flux at steady state as measured on the oxidation side of the sample in a Devanathan-Stachurski cell
J_(t)_	Time-dependent atomic hydrogen permeation flux as measured on the oxidation side of the sample in a Devanathan-Stachurski cell
K_Jc_	Stress Intensity Factor
K_JcH_	Stress Intensity Factor in hydrogen
NG	Natural Gas
OCTG	Oil Country Tubular Goods
RA	Reduction Area
RA_H_	Reduction Area with hydrogen
RA_air_	Reduction Area in inert environment
RRA	Relative Reduction Area
SMYS	Specified Minimum Yield Strength
SEB	Single Edge Bend
t_lag_	Time to achieve a value of J_(t)_/J_SS_ = 0.63
YS	Yield Strength
UTS	Ultimate Tensile Strength

## References

[1] (2018). European Parliament Directive (EU) 2018/2001 of the European Parliament and of the Council of 11 December 2018 on the promotion of the use of energy from renewable sources (recast). Off. J. Eur. Union.

[2] Puertas R., Marti L. (2022). Renewable energy production capacity and consumption in Europe. Sci. Total Environ..

[3] (2022). Gas infrastructure—Consequences of Hydrogen in the Gas Infrastructure and Identification of Related Standardisation Need in the Scope of CEN/TC 234.

[4] Ewan B.C.R., Allen R.W.K. (2005). A figure of merit assessment of the routes to hydrogen. Int. J. Hydrogen Energy.

[5] Hayden L., Douglas S. Hydrogen piping and pipeline code design rules and their interaction with pipeline material concerns, issues and research. Proceedings of the ASME 2009 Pressure Vessels and Piping Division Conference.

[6] Duprez L., Leunis E., Güngör Ö.E., Claessens S., Gangloff R.P., Somerday B.P. (2012). Hydrogen embrittlement of high strength, low alloy (HSLA) steels and their welds. Gaseous Hydrogen Embrittlement of Materials in Energy Technologies.

[7] (2023). Hydrogen Piping and Pipelines.

[8] Matos C.R., Carneiro J.F., Silva P.P. (2019). Overview of Large-Scale Underground Energy Storage Technologies for Integration of Renewable Energies and Criteria for Reservoir Identification. J. Energy Storage.

[9] Osman A.I., Mehta N., Elgarahy A.M., Hefny M., Al-Hinai A., Al-Muhtaseb A.H., Rooney D.W. (2022). Hydrogen production, storage, utilisation and environmental impacts: A review. Environ. Chem. Lett..

[10] Jahanbakhsh A., Potapov-Crighton A.L., Mosallanezhad A., Kaloorazi N.T., Maroto-Valer M.M. (2024). Underground hydrogen storage: A UK perspective. Renew. Sustain. Energy Rev..

[11] Xu K. (2012). Hydrogen embrittlement of carbon steels and their welds. Gaseous Hydrogen Embrittlement of Materials in Energy Technologies: The Problem, Its Characterisation and Effects on Particular Alloy Classes.

[12] Nanninga N.E. (2012). Fatigue crack initiation and fatigue life of metals exposed to hydrogen. Gaseous Hydrogen Embrittlement of Materials in Energy Technologies: The Problem, Its Characterisation and Effects on Particular Alloy Classes.

[13] Stalheim D., Bogges T., Marchi C.S., Somerday B., Boggess T., Jansto S. Microstructure and Mechanical Property Performance of Commercial Grade API. Proceedings of the 8th International Pipeline Conference.

[14] Somerday B.P., Marchi C.S., Nibur K.A., Stalheim D.G., Boggess T., Jansto S. Pipeline Steels in Gaseous Hydrogen. Proceedings of the ASME Pressure Vessels and Piping Division Conference.

[15] Nanninga N., Slifka A., Levy Y., White C. (2010). A review of fatigue crack growth for pipeline steels exposed to hydrogen. J. Res. Natl. Inst. Stand. Technol..

[16] Slifka A.J., Drexler E.S., Amaro R.L., Hayden L.E., Stalheim D.G., Lauria D.S., Hrabe N.W. (2018). Fatigue measurement of pipeline steels for the application of transporting gaseous hydrogen. J. Press. Vessel. Technol. Trans. ASME.

[17] Amaro R.L., Rustagi N., Drexler E.S., Slifka A.J. (2014). Sensitivity analysis of fatigue crack growth model for API steels in gaseous hydrogen. J. Res. Natl. Inst. Stand. Technol..

[18] Briottet L., Moro I., Lemoine P. (2012). Quantifying the hydrogen embrittlement of pipeline steels for safety considerations. Int. J. Hydrogen Energy.

[19] (2023). DVGW Project SyWeSt H2—Investigation of Steel Materials for Gas Pipelines and Plants for Assessment of their Sustainability with Hydrogen. https://www.dvgw.de/medien/dvgw/forschung/berichte/g202006-sywesth2-steel-dvgw.pdf.

[20] Cheng X.Y., Zhang H.X. (2020). A new perspective on hydrogen diffusion and hydrogen embrittlement in low-alloy high strength steel. Corros. Sci..

[21] American Petroleum Institute (2011). API Spec 5CT.

[22] (2015). Test Methods and Definitions for Mechanical Testing of Steel Products.

[23] (2020). Test Method for Measurement of Fracture Toughness.

[24] (2014). Method of Measurement of Hydrogen Permeation and Determination of Hydrogen Uptake and Transport in Metals by an Electrochemical Technique.

[25] Chai M., Song Y., Zhang Z., Duan Q., Cheng G. Effect of Hydrogen on Fracture Toughness Behavior of 2.25Cr-1Mo-0.25V Steel. Proceedings of the ASME 2018 Pressure Vessels and Piping Conference.

[26] Kim W.K., Koh S.U., Yang B.Y., Kim K.Y. (2008). Effect of environmental and metallurgical factors on hydrogen induced cracking of HSLA steels. Corros. Sci..

[27] Zhao Y., Seok M.-Y., Choi I.-C., Lee Y.-H., Park S.-J., Ramamurty U., Suh J.-Y., Jang J.-I. (2015). The role of hydrogen in hardening/softening steel: Influence of the charging process. Scr. Mater..

[28] Zhang T., Chu W.Y., Gao K.W., Qiao L.J. (2003). Study of correlation between hydrogen-induced stress and hydrogen embrittlement. Mater. Sci. Eng. A.

[29] Wu X.Q., Kim I.S. (2003). Effects of strain rate and temperature on tensile behavior of hydrogen-charged SA508 Cl.3 pressure vessel steel. Mater. Sci. Eng..

[30] Hoyos J.J., Masoumi M., Pereira V.F., Tschiptschin A.P., Paes M.T.P., Avila J.A. (2019). Influence of hydrogen on the microstructure and fracture toughness of friction stir welded plates of API 5L X80 pipeline steel. Int. J. Hydrogen Energy.

[31] Dong C.F., Liu Z.Y., Li X.G., Cheng Y.F. (2009). Effects of hydrogen-charging on the susceptibility of X100 pipeline steel to hydrogen-induced cracking. Int. J. Hydrogen Energy.

[32] Paterlini L., Casanova L., Ormellese M., Re G. (2023). Electrochemical Hydrogen Charging, Comparison of Different Methodologies. Structural.

[33] Bolzoni F., Paterlini L., Casanova L., Ormellese M. (2023). Hydrogen charging of carbon and low alloy steel by electrochemical methods. J. Appl. Electrochem..

[34] Verbeken K. (2012). Analysing hydrogen in metals: Bulk thermal desorption spectroscopy (TDS) methods. Gaseous Hydrogen Embrittlement of Materials in Energy Technologies: Mechanisms, Modelling and Future Developments.

[35] Wang R. (2009). Effects of hydrogen on the fracture toughness of a X70 pipeline steel. Corros. Sci..

[36] Ohtsubo T., Goto S., Amano M. (1985). Development Diffusible of Apparatus Hydrogen in Steel. Trans. ISIJ.

[37] Darling H.E. (1964). Conductivity of Sulfuric Acid Solutions. J. Chem. Eng. Data.

[38] Álvarez G., Peral L.B., Rodríguez C., García T.E., Belzunce F.J. (2019). Hydrogen embrittlement of structural steels: Effect of the displacement rate on the fracture toughness of high-pressure hydrogen pre-charged samples. Int. J. Hydrogen Energy.

[39] Cupertino-Malheiros L., Oudriss A., Thébault F., Piette M., Feaugas X. (2023). Hydrogen Diffusion and Trapping in Low-Alloy Tempered Martensitic Steels. Metall. Mater. Trans. A Phys. Metall. Mater. Sci..

[40] Fumagalli G., Bolzoni F., Fallahmohammadi E., Re G., Lazzari L. (2015). Electrochemical methods for determining diffusion coefficient of hydrogen in steels. Corros. Eng. Sci. Technol..

[41] Fallahmohammadi E., Bolzoni F., Fumagalli G., Re G., Benassi G., Lazzari L. (2014). Hydrogen diffusion into three metallurgical microstructures of a C-Mn X65 and low alloy F22 sour service steel pipelines. Int. J. Hydrogen Energy.

[42] Trautmann A., Mori G., Oberndorfer M., Bauer S., Holzer C., Dittmann C. (2020). Hydrogen uptake and embrittlement of carbon steels in various environments. Materials.

[43] Truschner M., Trautmann A., Mori G. (2021). The Basics of Hydrogen Uptake in Iron and Steel. BHM Berg- und Hüttenmännische Monatshefte.

[44] Trautmann A., Mori G., Loder B. (2021). Hydrogen Embrittlement of Steels in High Pressure H_2_ Gas and Acidified H_2_S-saturated Aqueous Brine Solution. BHM Berg- und Hüttenmännische Monatshefte.

[45] Nguyen T.T., Heo H.M., Park J., Nahm S.H., Beak U.B. (2021). Stress concentration affecting hydrogen-assisted crack in API X70 pipeline base and weld steel under hydrogen/natural gas mixture. Eng. Fail. Anal..

[46] Moro I., Briottet L., Lemoine P., Andrieu E., Blanc C., Odemer G. (2010). Hydrogen embrittlement susceptibility of a high strength steel X80. Mater. Sci. Eng. A.

[47] Duncan A., Lam P.S., Adams T. Tensile testing of carbon steel in high pressure hydrogen. Proceedings of the ASME 2007 Pressure Vessels and Piping Conference.

[48] Nanninga N.E., Levy Y.S., Drexler E.S., Condon R.T., Stevenson A.E., Slifka A.J. (2012). Comparison of hydrogen embrittlement in three pipeline steels in high pressure gaseous hydrogen environments. Corros. Sci..

[49] Nanninga N., Levy Y., Drexler E., Condon R., Stevenson A., Slifka A. Tensile Behavior of Pipeline Steels in High Pressure Gaseous Hydrogen Environments. https://tsapps.nist.gov/publication/get_pdf.cfm?pub_id=907912.

[50] Meng B., Gu C., Zhang L., Zhou C., Li X., Zhao Y., Zheng J., Chen X., Han Y. (2017). Hydrogen effects on X80 pipeline steel in high-pressure natural gas/hydrogen mixtures. Int. J. Hydrogen Energy.

[51] Nguyen T.T., Tak N., Park J., Nahm S.H., Beak U.B. (2020). Hydrogen embrittlement susceptibility of X70 pipeline steel weld under a low partial hydrogen environment. Int. J. Hydrogen Energy.

[52] (2014). Test Methods for Evaluating Material Compatibility in Compressed Hydrogen Applications—Metals.

[53] Svein E., Liane S. (2009). EFC—Guidelines on Materials Requirements for Carbon and Low Alloy Steels for H_2_S-Containing Environments in Oil and Gas Production.

